# Short-Term Effects of Changing Precipitation Patterns on Shrub-Steppe Grasslands: Seasonal Watering Is More Important than Frequency of Watering Events

**DOI:** 10.1371/journal.pone.0168663

**Published:** 2016-12-20

**Authors:** Justine A. Densmore-McCulloch, Donald L. Thompson, Lauchlan H. Fraser

**Affiliations:** 1 Department of Natural Resource Sciences, Thompson Rivers University, Kamloops, British Columbia, Canada; 2 Agriculture and Agri-Food Canada, Lethbridge, Alberta, Canada; University of Saskatchewan, CANADA

## Abstract

Climate change is expected to alter precipitation patterns. Droughts may become longer and more frequent, and the timing and intensity of precipitation may change. We tested how shifting precipitation patterns, both seasonally and by frequency of events, affects soil nitrogen availability, plant biomass and diversity in a shrub-steppe temperate grassland along a natural productivity gradient in Lac du Bois Grasslands Protected Area near Kamloops, British Columbia, Canada. We manipulated seasonal watering patterns by either exclusively watering in the spring or the fall. To simulate spring precipitation we restricted precipitation inputs in the fall, then added 50% more water than the long term average in the spring, and vice-versa for the fall precipitation treatment. Overall, the amount of precipitation remained roughly the same. We manipulated the frequency of rainfall events by either applying water weekly (frequent) or monthly (intensive). After 2 years, changes in the seasonality of watering had greater effects on plant biomass and diversity than changes in the frequency of watering. Fall watering reduced biomass and increased species diversity, while spring watering had little effect. The reduction in biomass in fall watered treatments was due to a decline in grasses, but not forbs. Plant available N, measured by Plant Root Simulator (PRS)-probes, increased from spring to summer to fall, and was higher in fall watered treatments compared to spring watered treatments when measured in the fall. The only effect observed due to frequency of watering events was greater extractable soil N in monthly applied treatments compared to weekly watering treatments. Understanding the effects of changing precipitation patterns on grasslands will allow improved grassland conservation and management in the face of global climatic change, and here we show that if precipitation is more abundant in the fall, compared to the spring, grassland primary productivity will likely be negatively affected.

## Introduction

In grassland ecosystems, water availability is the major limiting factor to primary production [1]; therefore changes in precipitation patterns and increased risk of drought will likely have a major impact on grassland ecosystems [2,3]. A key prediction of altered precipitation is an increased risk of drought [3], and yet few studies have investigated the effect of potential changes in precipitation amount and distribution on terrestrial ecosystems [4–6]. Predicted changes in precipitation vary across grasslands globally (increase, no change, decrease) but all models agree that precipitation will be more variable with more extreme drought events [5]. It is important to understand how changes in precipitation will impact grasslands because of the important services they provide, such as rangeland for livestock [7–9], biodiversity ‘hotspots’ [10], as well as providing food, forage fuel, medicines, shelter, habitat, climate regulation, cultural and religious sites, and recreation activities [11].

Changing the frequency and intensity of precipitation events can affect soil water availability in grasslands. Increasing rainfall event size while decreasing frequency in grassland ecosystems may lead to small increases in plant-available soil water during rainfall pulses, while increasing the length and intensity of drought in the interpulse period [12–15]. Knapp et al. [12] found altering rainfall events from ambient so that they were less frequent but supplied, overall, the same quantity of water, increased plant species diversity but reduced aboveground net primary productivity in a native grassland ecosystem in north-east Kansas, United States. Decreased frequency of water pulses have been shown to cause shifting dominance from fast growing to slow growing grass species as the interpulse period increases [16]. Therefore, the length of the interpulse period may have a significant effect on plant dominance and species diversity [17]. What is not known is whether frequency is more or less important than seasonal timing (e.g., spring or fall) of precipitation events. In addition to species-specific drought tolerance traits, nitrogen dynamics in soil can also be affected by water pulses.

The majority of nitrogen in natural environments comes from nitrogen fixation activity of bacteria, both free-living and in symbiosis with plants; other sources are human-mediated industrial fixation, and wet and dry atmospheric deposition [18]. Nitrogen mineralization is tied to water availability within soil. Bacterial population growth increases rapidly after rewetting dry soil, to growth rates and respiration levels higher than soils kept constantly moist. Iovieno and Bååth [19] showed that approximately four days after a wetting pulse, bacterial growth rates returned to the similar bacterial growth rate observed in constantly moist soils. This increased activity during a water pulse can cause a 25–30% increase in overall bacterial growth, compared to constantly moist soils [19].

Environmental gradients, such as precipitation, soil, latitude, and altitude, are important in structuring plant communities [20]. Consideration of environmental gradients is therefore an important aspect of understanding biological responses to climate change [21]. As there is rarely an opportunity to study plant community changes over time scales exceeding a decade, gradients can be used to interpret how plant communities may change in the long-term [21]. Understanding the effects of changing precipitation patterns on the grassland plant community will allow improved grassland conservation and management in the face of global climate change.

We tested the effects of altered precipitation patterns along a natural primary productivity gradient. We asked the following questions (a) How does nitrogen availability change with seasonality and frequency of precipitation? (b) How do variation in seasonality and frequency of precipitation events affect grassland community productivity and plant species diversity? (c) How do precipitation patterns affect the relative abundance of grasses and forbs?

## Materials and Methods

### Study Site

This study was conducted under permit #102724 from the British Columbia Ministry of Environment, Parks and Protected Areas Division. Our study was carried out in Lac du Bois Grassland Protected Area (GPA), British Columbia, Canada. Lac du Bois GPA is 15 000 ha, moderately grazed by cattle and classified as a shrub-steppe grassland [11,22]. Three sites were selected for study along an elevational gradient that positively corresponded with primary productivity: a lower, middle and upper grassland site.

The lower grassland was located at 580 m asl (meters above sea level) (NAD 83, 10U 680869E 5622735N). Soil at this site is classified as Brown Chernozem [23]. Vegetation at the site is dominated by the grass *Pseudoroegneria spicata* (Pursh) A. Love, *Poa secunda* ssp. *secunda* J. Presl, and the woody shrub *Artemisia tridentata* Nutt. The middle grassland site was 755 m asl (NAD 83, 10U 0680873E, 5625967N) and the soil classified as Dark Brown Chernozem [23]. Species richness at the middle grassland is higher than the lower elevation site, and dominated by *Pseudoroegneria spicata* and *Poa secunda* ssp. *secunda*. The upper grassland at 900 m asl (NAD 83, 10U 0679866E, 5629464N) has Black Chernozem soil [23], and the plant community is typically dominated by *Festuca campestris* Rydb., *Achnatherum richardsonii* (Link) Barkworth and *Hesperostipa comata* (Trin. & Rupr.) Barkworth.

The region is semi-arid with annual precipitation of 279 mm, 75.5 mm of which is snowfall [23]. A slight increase in precipitation occurs with elevation, which corresponds to changes in soil type, plant community, and increasing productivity with elevation of the sites [23,24].

### Experimental design

In April 2010, one cattle exclosure (approximately 30 m x 30 m) was erected at each of the three sites. Experimental plots within the exclosures were set up and treatments started May 2010. Plots were 1 x 1 m square with an average of 1 m between plots (minimum of 0.5 m). Sagebrush (*Artemisia tridentata*) plants were deliberately avoided. All sites were in areas grazed by cattle in previous years.

Hand watering was applied to study the effects of precipitation change on the grassland plant community in 2010 and 2011. All watering treatment plots were covered with temporary rainout shelters (RS) in both spring (May-June) and fall (September-October), to block the majority of natural precipitation to fall directly on the watering treatment plots. Unmanipulated ambient precipitation (control) plots were not covered by RS’s. The RS’s were removed from plots during July and August, and were similar in design to Kochy & Wilson [25] and Carlyle et al. [22]. Previous work in the same grasslands, with the same RS design, showed that plots with a RS had an approximate 50% reduction in soil moisture compared to ambient [22]. The shelter consisted of a thin sheet of plastic (Tufflite IV™ 6mil Polyethylene film, Tyco Plastics and Agricultural Films Monroe, LA, USA) attached to four wooden stakes at the corners of the plot. The plastic was anchored at 1 m height on the north-west side of the plot, and at 30 cm on the other three corners. Aside from the unmanipulated control plots, four combinations of watering treatments were applied by hand watering: 1) spring watering applied every week (spring-frequent), 2) spring watering applied every four weeks (spring-intensive), 3) fall watering applied every week (fall-frequent), 4) fall watering applied every four weeks (fall-intensive). The water addition amount was a 50% increase in the historical 30-year average for the respective month of watering (Table 1); therefore, the spring watering treatment plots received a total addition of 89.4 mm, which was more than the fall watering treatment plots which received a total of 66.3 mm. Relative rather than absolute watering manipulations are recommended for field precipitation experiments [26]. Water addition was applied slowly to minimize surface runoff. All treatments were applied in a fully factorial design, creating 5 treatment and control plots in a block. Blocks were replicated six times at each of three sites to control for spatial heterogeneity within a site. Treatments were randomly assigned to plots within each block.

**Table 1:** Water amounts added to plots for the 1 week and 4 week treatments in the respective month of addition. No water was added during July and August. Average precipitation was based on historical (1971–2000) data [27].

	SPRING		SUMMER		FALL	
	May	June	July	August	September	October
**Average precipitation (mm)**	24.4	35.2	NA	NA	28	16.2
**50% increase (mm)**	36.6	52.8	NA	NA	42	24.3
**Amount per 1 week (L)**	9.15	13.2	NA	NA	10.5	6.075
**Amount per 4 weeks (L)**	36.6	52.8	NA	NA	42	24.3

### Sampling

The sampling area was the centre 0.25 cm^2^ of each plot. This left a 25 cm border between the plot edge and sampling area to account for edge effects. In a similar study, Yahdjian & Sala [28] found edge effects extending up to 20 cm under the rainout shelters; therefore, the 25 cm border was considered an adequate buffer.

Soil moisture as volumetric water content (VWC) and temperature (°C) measurements were logged every half hour from May through October. Moisture and temperature probes were placed in one block at each of the three sites. Soil moisture probes (Soil Moisture Smart Sensor, S-SMB-M005 using an ECH2O® Dielectric Aquameter probe, Decagon Devices, Inc.) were 10 cm long and placed vertically into the soil. Measurements were averaged over the length of the probe. The probes were connected to a HOBO® Micro Station data logger or Weather Station data logger, Onset Computer Corporation. Soil moisture data were calibrated for soil type as in Carlyle et al. [22]. Soil temperature probes (TMC50-HD, connected to a HOBO® U12 Data Logger, Onset Computer Corporation) were placed approximately 5 cm below the soil surface. Two of the soil probes in the upper grassland malfunctioned, and so we had no data in the spring-intensive treatment past August 18, 2011 nor the fall-frequent treatment past June 30, 2011.

Plant-available nitrogen (N) was measured using Plant Root Simulator (PRS)-probes [29] placed in two replicate blocks per site. These probes consisted of an ion-replacing membrane held in a plastic frame (15 cm x 2.5 cm x 0.5 cm) that captured free ions in the soil. N forms of interest were nitrate (NO3-) and ammonium (NH4+). Two probes were needed for each analysis–one that captured and replaced anions (NO3-), and one for cations (NH4+). Probes were replaced every sixty days, according to the three measurement seasons of spring, summer and fall. The three sets of probes allowed a continuous measurement of soil N dynamics over the entire six-month study season.

Total N in plot soils was also done to test the correlation between extractable NO3- and NH4+ to the relative amount of plant-available N measured by PRS-probes. Samples of the 0–15 cm soil surface were collected from the same plots as the probes in early November 2011, shortly after the final watering treatments were completed. Samples were air-dried then sieved using a 2 mm mesh. The samples were extracted for 1 hour at a ratio of 2.5 g soil: 25 ml 2N KCl and the centrifuged extracts analyzed for available NH4+- N and NO3—N using an OI-Analytical “Alpkem FSIV” segmented flow analyzer. Analysis was performed at the Technical Services Laboratory for the British Columbia Ministry of Environment in Victoria, BC, Canada.

In November 2011, aboveground biomass from the centre 0.25 m2 sampling area was clipped to soil surface, sorted to species, dried at 65°C for two days, then weighed.

### Statistical analysis

Soil moisture and temperature were analysed using a repeated-measures ANOVA. Study design allowed the use of General Linear Models (GLM), using site (lower, middle, upper), watering season (control, spring, fall) and watering frequency (ambient, weekly, monthly) as factors. Additionally, 3-way ANOVAs were used to test grassland type (lower, middle, upper), watering season (spring, fall) and watering frequency (weekly, monthly). Post-hoc Tukey’s HSD tests were used to determine significant differences between the means. Two types of soil N measurements were analysed, PRS™– probe N and soil extractable N. The PRS™ data had three measurement seasons; spring, summer, and fall. For plant community analyses, dependent variables included dry biomass, Shannon-Weiner diversity index and plant species richness. A functional group analysis was conducted on grasses and forbs. Data were either natural log +1 transformed or square root transformed to meet the ANOVA assumption of equality of variances, which resulted in normalized distributions for the data. All analyses were performed using SYSTAT (version 13). All soil N and plant community data are available as Supporting Information files (S1 File).

## Results

### Soil Moisture and Temperature

**Fig 1:**
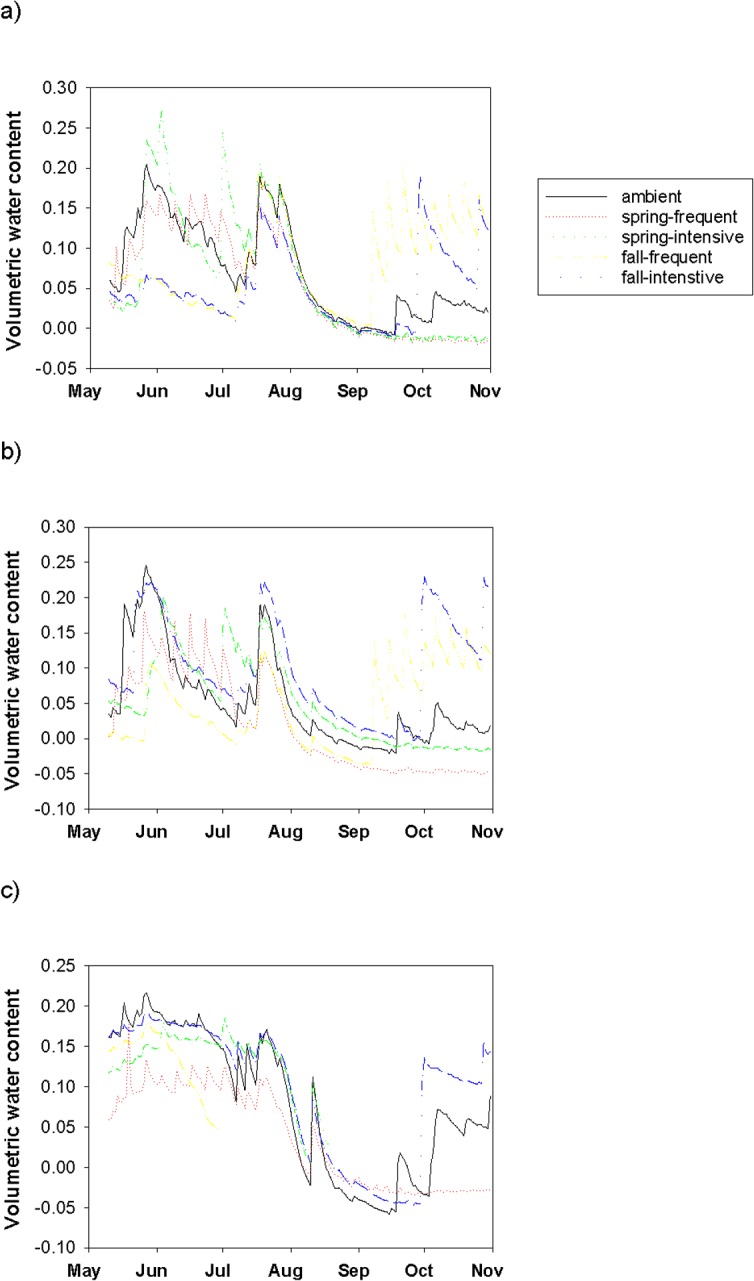
Lower grassland volumetric water content (VWC). VWC in ambient (control) and watering treatment plots (spring/ fall, weekly/ monthly).

All grassland sites (lower, middle and upper) showed similar effects of the watering treatment (Fig 1). The VWC daily mean for the fall frequent watering treatment was higher than the other watering plots (F = 11.290, df = 3, P = 0.009). Spring, summer and fall seasons were also analysed separately. Daily mean VWC was affected by watering in all three seasons. In the spring, fall watered plots were drier than either spring watered or ambient (F = 15.942, df = 3, P = 0.004). During the summer, spring frequent plots and fall intensive plots were drier than the others (F = 7.614, df = 3, P = 0.022). During the fall, spring watered plots were driest (F = 85.097, df = 3, P ≤ 0.001).

Daily mean temperature was was analysed. We found no effect of watering on temperature by site or by season; however, we did find that soil temperatures were lower directly following watering events (F = 18.230, df = 3, P = 0.020).

### Nitrogen

**Fig 2:**
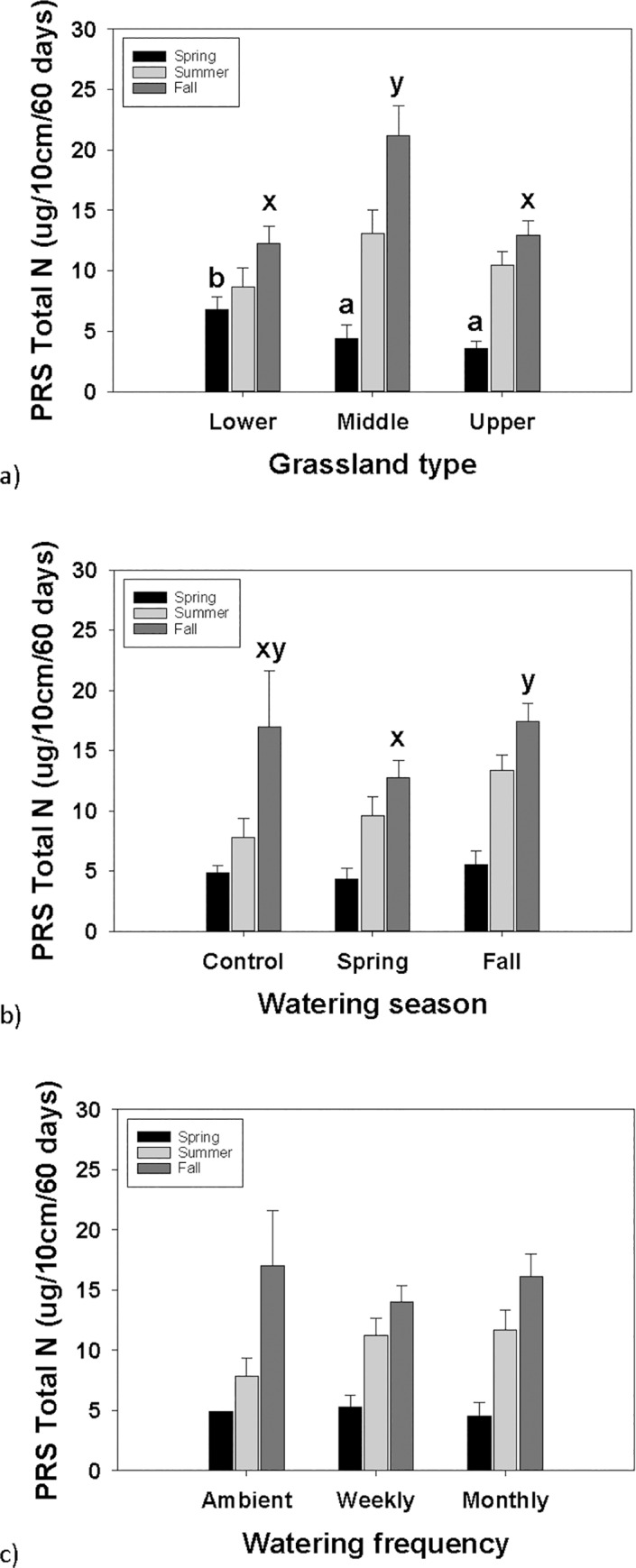
PRS–probe total N amounts. Total N by (a) grassland type (lower, middle and upper), (b) watering season (control, spring and fall), and (c) watering frequency (ambient, weekly and monthly), measured at three separate intervals: spring, summer and fall. Error bars are + SE, bars sharing similar letters are not significantly different by measurement in spring, summer and fall. If there are no letters the model was not significant.

GLMs on the PRS™– probe data showed similar trends between total N, NH3 and NO4; therefore only the total N is presented. Three GLM analyses were conducted, and three 3-way ANOVAs, for spring, summer and fall. In spring, PRS™– total N was affected by site (Table 2), such that the lower grassland site had higher total N than the middle and upper sites (Fig 2A). Spring total N was not affected by watering season or frequency (Table 2). In summer there was no difference in PRS™– total N between treatments (Table 2). In fall, site and watering frequency affected PRS™– total N (Table 2). By site, the middle grassland had a greater total N compared to the lower and upper grassland sites (Fig 2A). By watering season, the fall watering had a greater total N than the spring watering in the fall season (Fig 2B). Water frequency did not affect PRS™– total N (Table 2; Fig 2C). There were no significant interactions between factors. Overall, there was a trend towards increasing PRS™– total N from spring to summer to fall (Fig 2).

**Table 2:** Three 3-way ANOVAs (Spring, Summer and Fall) to determine the effect of grassland site, watering season and watering frequency on PRS™ –probe total N amounts. Bold values are significant at p < 0.05, while italicized values are significant at p < 0.10.

SOURCE	Degrees Freedom	Mean Squares	F-Ratio	p-Value
**SPRING**				
**Grassland site (G)**	**2**	**41.134**	**5.373**	**0.022**
Watering season (WS)	1	8.640	1.129	0.309
Watering frequency (WF)	1	3.682	0.481	0.501
G x WS	2	10.501	1.372	0.291
*G x WF*	*2*	*25*.*303*	*3*.*305*	*0*.*072*
*WS x WF*	*1*	*26*.*042*	*3*.*402*	*0*.*090*
G x WS x WF	2	6.915	0.903	0.431
Error	12	7.656		
**SUMMER**				
Grassland site (G)	2	38.018	1.600	0.242
*Watering season (WS)*	*1*	*82*.*882*	*3*.*489*	*0*.*086*
Watering frequency (WF)	1	1.215	0.051	0.825
G x WS	2	40.900	1.722	0.220
G x WF	2	25.816	1.087	0.368
WS x WF	1	55.207	2.324	0.153
G x WS x WF	2	10.025	0.422	0.665
Error	12	23.756		
**FALL**				
Grassland site (G)	2	**84.990**	**4.301**	**0.039**
Watering season (WS)	1	**132.540**	**6.707**	**0.024**
Watering frequency (WF)	1	26.042	1.318	0.273
G x WS	2	8.574	0.434	0.658
G x WF	2	2.995	0.152	0.861
WS x WF	1	18.375	0.930	0.354
G x WS x WF	2	49.639	2.512	0.123
Error	12	19.760		

**Fig 3:**
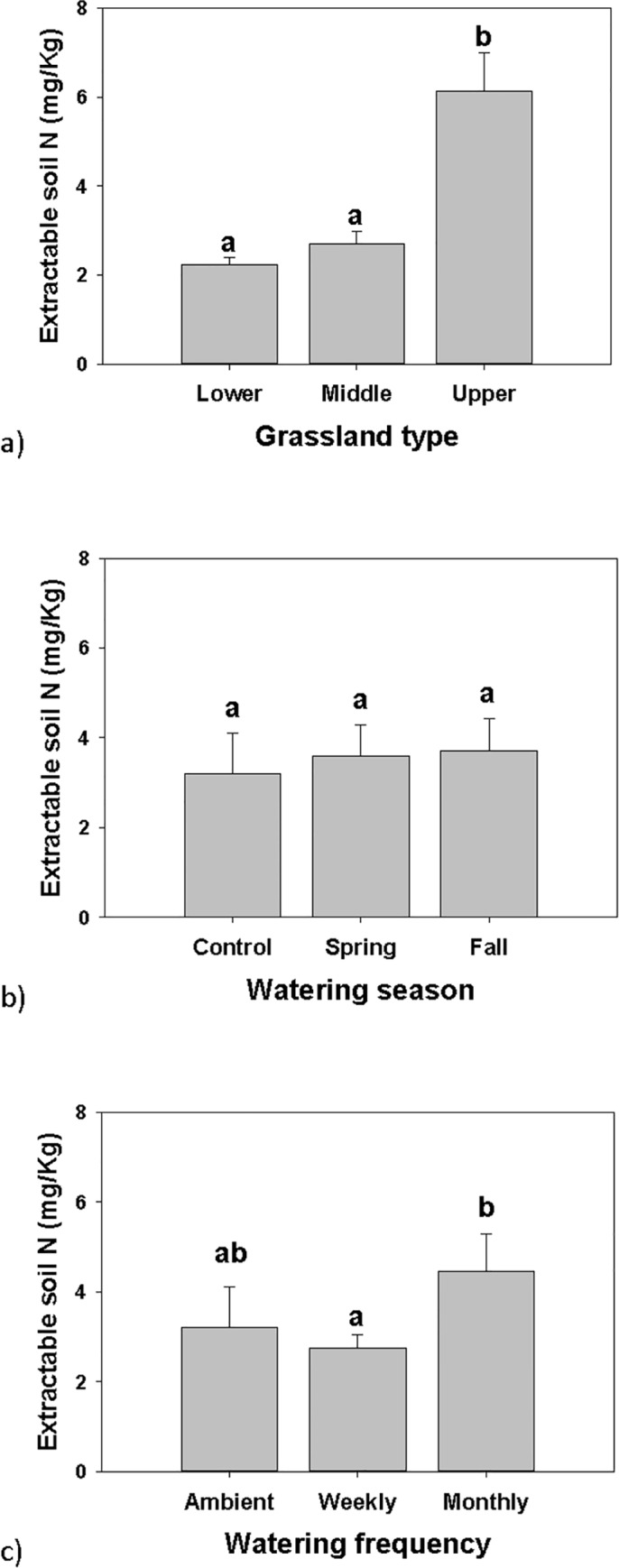
Extractable soil N. N amounts by (a) grassland type (lower, middle and upper), (b) watering season (control, spring and fall), and (c) watering frequency (ambient, weekly and monthly), measured in the fall. Error bars are + SE, bars sharing similar letters are not significantly different by measurement in spring, summer and fall.

The extractable soil N showed similar trends between total N, NO3 and NH4, and so only total N is presented. A GLM of extractable total N showed an effect by site (F = 19.460, df = 2, P <0.001) and watering frequency (F = 3.463, df = 1, P = 0.077). The upper grassland had the greatest total N (Fig 3A). Monthly watering had a greater total N compared to weekly watering (Fig 3C). Watering season did not affect extractable total N (Fig 3B). A 3-way ANOVA showed an effect by site only, and there were no significant interactions between factors (Table 3).

**Table 3:** General Linear Model to determine the effect of grassland site, watering season and watering frequency on Extractable soil N. Bold values are significant at p < 0.05.

**Grassland site (G)**	**2**	**21.576**	**19.624**	**<0.001**
Watering season (WS)	1	1.767	1.607	0.411
Watering frequency (WF)	1	4.650	2.555	0.136
G x WS	2	0.172	0.156	0.857
G x WF	2	2.739	2.491	0.124
WS x WF	1	0.924	0.841	0.377
G x WS x WF	2	1.060	0.964	0.409
Error	12	1.099		

## Plant Community

**Fig 4:**
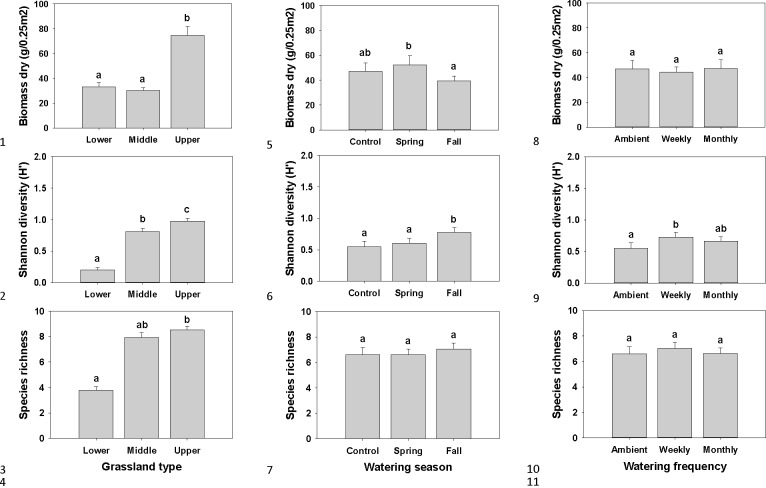
Plant community results. Dry plant biomass by (a) grassland type (lower, middle and upper), (b) watering season (control, spring and fall), and (c) watering frequency (ambient, weekly and monthly). Shannon diversity index by (a) grassland type, (b) watering season, and (c) watering frequency. Species richness by (a) grassland type, (b) watering season, and (c) watering frequency. All measured in the fall. Error bars are + SE, bars sharing similar letters are not significantly different by measurement in spring, summer and fall.

GLMs and 3-way ANOVAs showed that plant biomass was affected by site and by watering season, but not by watering frequency (Table 4). The upper grassland site had the greatest biomass (Fig 4A). Spring watering resulted in a greater plant biomass compared to fall watering (Fig 4B). Species diversity, as measured by the Shannon diversity index, was affected by site, watering season and watering frequency (Table 4). The upper grassland had the highest diversity index and the lower grassland had the lowest (Fig 4D). Fall watering resulted in the highest diversity index, compared to control and spring watering (Fig 4E). The weekly watering treatment had a higher biomass than the ambient treatment (Fig 4F). Species richness was only affected by site, not by watering season or frequency (Table 4). The upper grassland had more species than the lower grassland (Fig 4G). There were no significant interactions between factors.

**Table 4:** Three 3-way ANOVAs to determine the effect of grassland site, watering season and watering frequency Biomass, Shannon Diversity and Species Richness. Bold values are significant at p < 0.05, italicized at p < 0.10.

SOURCE	Degrees Freedom	Mean Squares	F-Ratio	p-Value
**BIOMASS**				
**Grassland site (G)**	**2**	**6.998**	**30.448**	**<0.001**
*Watering season (WS)*	*1*	*0*.*692*	*3*.*009*	*0*.*088*
Watering frequency (WF)	1	0.042	0.184	0.669
G x WS	2	0.012	0.053	0.949
G x WF	2	0.079	0.345	0.710
WS x WF	1	0.004	0.019	0.890
G x WS x WF	2	0.304	1.325	0.274
Error	59	0.230		
**SHANNON DIVERSITY**				
**Grassland site (G)**	**2**	**3.870**	**59.592**	**<0.001**
**Watering season (WS)**	**1**	**0.607**	**9.352**	**0.003**
Watering frequency (WF)	1	0.101	1.548	0.218
G x WS	2	0.094	1.451	0.243
G x WF	2	0.094	1.446	0.244
WS x WF	1	0.008	0.121	0.729
G x WS x WF	2	0.068	1.022	0.366
Error	24	0.065		
**SPECIES RICHNESS**				
**Grassland site (G)**	**2**	**169.872**	**57.501**	**<0.001**
Watering season (WS)	1	5.574	1.887	0.175
Watering frequency (WF)	1	4.525	1.532	0.221
G x WS	2	1.570	0.532	0.590
G x WF	2	0.667	0.226	0.799
WS x WF	1	0.525	0.178	0.675
G x WS x WF	2	0.667	0.226	0.799
Error	24	2.954		

### Plant Functional Groups

**Fig 5:**
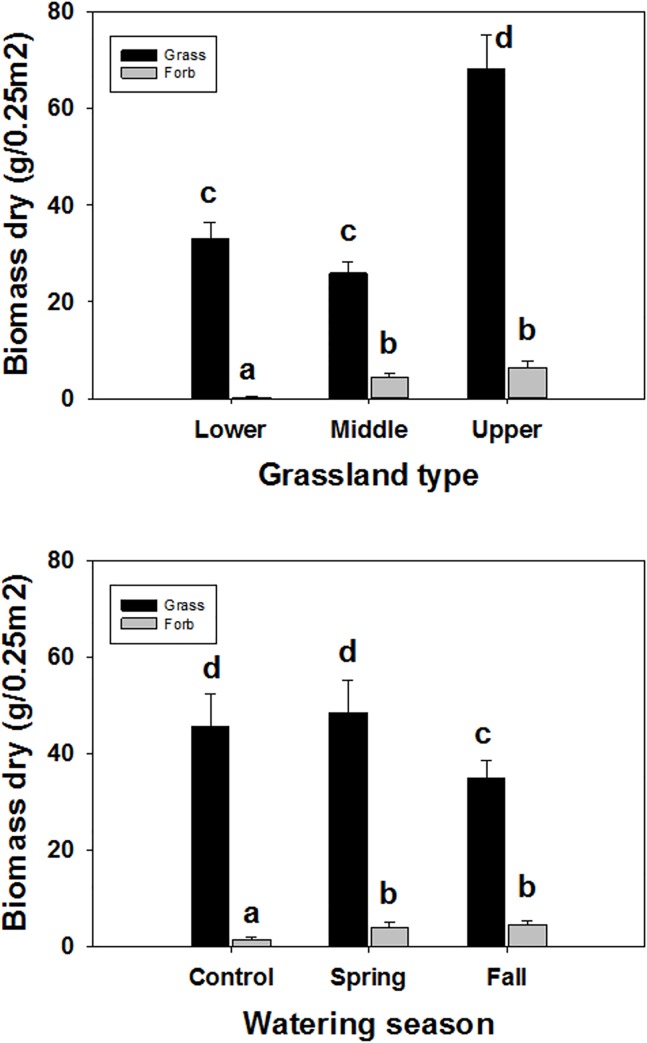
Functional group response. Dry plant biomass of grasses and forbs by (a) grassland type (lower, middle and upper), and (b) watering season (control, spring and fall). Error bars are + SE, bars sharing similar letters are not significantly different by measurement in spring, summer and fall.

A GLM determined that the functional groups, grasses and forbs, differed by biomass (Table 5). We found that functional group and site were significant main effects (Table 5). We also found interacting effects between functional group and site, and functional group and watering season (Table 6). Grasses, in general, had much greater biomass than forbs. The upper grassland had greater biomass of grasses than the lower and middle grassland sites (Fig 5A), while the upper and middle grassland site had greater biomass of forbs than the lower grassland (Fig 5A). Spring watering, and ambient, plots had greater biomass of grasses compared to fall watering (Fig 5B). Fall and spring watering increased the forb biomass compared to the watering control (Fig 5B).

**Table 5:** General Linear Model to determine the effect of functional group, grassland site, watering season and watering frequency on dry biomass. Bold values are significant at p < 0.05.

SOURCE	Degrees Freedom	Mean Squares	F-Ratio	p-Value
**Functional group**	**1**	**66,679.601**	**162.130**	**<0.001**
**Grassland site**	**2**	**9,128.882**	**22.198**	**<0.001**
*Watering season*	*1*	*1*,*418*.*187*	*3*.*448*	*0*.*065*
Watering frequency	2	42.231	0.103	0.902
Error	171	411.257		

**Table 6:** 3-way ANOVA to determine the effect of FG (functional group), Site (grassland site), and Season (watering season), and their interactions, on dry biomass. Bold values are significant at p < 0.05.

SOURCE	Degrees Freedom	Mean Squares	F-Ratio	p-Value
**FG**	**1**	**62,842.208**	**190.177**	**<0.001**
**Site**	**2**	**7,408.670**	**22.421**	**<0.001**
Season	2	692.050	2.094	0.127
**FG x Site**	**2**	**5,435.599**	**16.450**	**<0.001**
*FG x Season*	*2*	*982*.*635*	*2*.*974*	*0*.*054*
Site x Season	4	383.651	1.161	0.330
FG x Site x Season	4	257.439	0.779	0.540
Error	160	330.440		

## Discussion

We found that altering the season of watering affected plant biomass, diversity and plant available N. Whereas the frequency of watering events seemed to have little effect on grassland shrub-steppe communities. The reduction in biomass as a result of fall watering compared to spring watering seems to be a result of a decline in grass biomass, rather than forb biomass.

The watering treatments affected soil VWC. Welker et al. [30] found that water from small precipitation events (less than 3 mm) caused by natural rain showers or by irrigation had approximately the same residence time in soil. For larger events (above 6 mm) the irrigated water addition stayed significantly longer than water from natural rainfalls. Water from precipitation events < 10 mm remained in the soil for about two days, while water from those > 10 mm stayed consistently longer. In our experiment, the smallest water addition applied was 6.075 mm, so we can assume VWC was increased in the plots and persisted for a minimum of several days after each watering, as was seen in a similar study in the same area [22].

N availability, as measured by PRS™– probes, tended to increase with each successive season of measurement, from spring to summer to fall. This result is contrary to previous research on N availability in a Californian grassland to shrubland gradient, where N availability decreased as the growing season progressed [31]. In our study, the increase in available N in the fall may be due to a combination of increased ambient precipitation, watering treatments, and reduced plant growth, specifically grasses, during the fall growing season. These conditions may lead to a temporal decoupling of N availability and uptake as previously proposed [15,32,33]. Moist soil conditions provide the opportunity for microbes to mineralize labile N, but lower plant growth or fewer plants that are active in the fall make it likely that N uptake is lower leading to accumulation of N in the soil [34]. This reasoning would also explain why we found higher N availability in the PRS™– probes measured in the fall in plots treated with fall watering compared to spring watering. We are likely seeing increased bacterial growth in the fall, resulting in higher N availability, but a reduced ability of plants to acquire those nutrients because their growth rates are slowing as the plants begin to senesce.

Extracted N from soil samples show more than twice the total N in the upper grassland site compared to the lower and middle sites. Higher total extracted N reflects the more productivity grassland community found in the upper site compared to the lower and middle sites [22,24]. This alternate form of N measurement is a different way of estimating available N by extracting NO3- and NH4+ from a larger soil sample than the PRS™- probes, and is not dependent on water movement through soil. Why we found greater extractable N in soils watered intensively (monthly) compared to frequently (weekly) is somewhat puzzling because this was the only treatment effect on N that was observed due to watering frequency. Spence et al. [35] found that a 3-wk watering interval led to an increase in plant-available soil P compared to weekly watering, suggesting that changes in frequency can significantly affect nutrient cycling.

Spring watering increased plant production compared to fall watering, but not to the control plots. The difference between spring and fall watering was due to a reduction in grass growth, measured as biomass clipped in the fall, as a result of fall watering; or, rather, the delay in fall watering. Forb biomass, however, was similar between spring and fall watering treatments, but the controlled watering treatment had less biomass than the spring and fall watering treatments. Spring watering increased plant productivity in an arid sagebrush steppe in Oregon (USA) [36], but it took four years after the beginning of the experiment for this result to occur. Another watering experiment in a California grassland showed a rapid and positive biomass response to an extended spring rainy season [37]. In the California experiment, the strongest response to spring watering was by nitrogen-fixing forbs. Because forb biomass was increased in our spring watered plots compared to control plots we suspect that nitrogen-fixing forbs also responded to spring watering in our system. The fact that forb biomass in fall watered plots was more than the control plots is likely because grass biomass in the fall watered plots was significantly reduced, thus potentially releasing competitive dominance on the subordinate forbs.

Shifts in plant production and species composition between the spring and fall watered treatments had important consequences for biodiversity. With a reduction in biomass, as observed within the fall watered treatments, we found an increase in plant diversity, similar to watering experiments by Knapp et al. [12] and Fay et al. [38]. We suspect that the reduction in biomass released competitive pressure from dominant grasses, providing the opportunity for subordinate species to increase their relative proportional biomass [39]. Prevéy and Seastedt [40] also found that altered seasonality of precipitation can have direct effects on plant community composition in a grassland in Colorado, USA; such that increased winter precipitation caused an increase in cover of winter-active grasses and reduced species diversity. We did not see an increase in species richness with a biomass reduction, but the relative increase in forbs, in combination with a reduction in grasses, within the fall watered plots resulted in an increase in the Shannon diversity index. Since increased diversity, specifically functional diversity, may, in the long-term, affect ecosystem function and resistance to precipitation change [41, 42], it is possible that an extended fall rainy season may result in a system that is more resistant to a spring drought over time.

The majority of significant results we observed related to the seasonal application of watering, whether plots received spring or fall watering. Frequency of watering events, between frequent (weekly) and intensive (monthly) additions, had little effect. However, we did find that the frequent application of watering resulted in a higher species diversity than ambient conditions, and that intensive watered plots had higher extractable soil N than frequently watered plots. The fact that Spence et al. [35] found weekly watering of grasslands in a Mongolian steppe, compared to 3-wk watering, increased the total abundance of forbs suggests that more work is needed to disentangle the effects of frequency of rainfall events on grassland communities.

There are a number of climate change papers that show an immediate response to climate manipulations in grasslands [37,43,44,45], but others have demonstrated a delayed response from the plant community [6,36,46,47]. The study presented here is only short-term, so may not measure all changes that will occur with changing precipitation.

## Conclusion

After two years of field application, seasonal watering treatments altered the mean and variability of soil moisture, plant productivity, and plant diversity. The frequency of rainfall events had little impact on grassland nitrogen levels or plant community measurements. A reduction in growth by grasses under the fall watering treatment resulted in lower overall plant productivity and a concomitant increase in plant diversity. The fact that N availability in the soil is increased by fall watering suggests that there is the potential for N loss through leaching or denitrification. Climate factors play a large role in regulating rates of N mineralization and uptake, and the effects of these can change dramatically during a single growing season.

In terms of the impact of altered precipitation on forage productivity for the cattle industry, it would appear that an extended spring rainy season would result in the best outcome for sustainable range production. However, if climatic change resulted in a delayed fall rainy season there would be a reduction in forage productivity, at least in the short term. If plant available N is somehow retained in the system through the winter, it is possible that the long-term effects of fall watering might equilibrate to current ambient conditions.

## Supporting Information

S1 File Meta data, plant community, functional group, PRS probes N, soil extractable N. (XLSX)
